# PGE1 Suppresses the Expression of M2 Markers on Macrophages Through Prostaglandin Receptors

**DOI:** 10.3390/cells14241992

**Published:** 2025-12-15

**Authors:** Hiroyuki Tsuchiya, Takehiko Hanaki, Jun Yoshida, Mayu Obora, Yoshiyuki Fujiwara, Daisuke Nanba

**Affiliations:** 1 Division of Regenerative Medicine and Therapeutics, Department of Genomic Medicine and Regenerative Therapy, Faculty of Medicine, Tottori University, Yonago 680-8503, Japan; 2 Division of Gastrointestinal and Pediatric Surgery, Department of Surgery, Faculty of Medicine, Tottori University, Yonago 680-8503, Japan; 3 Division of Medical Education, Department of Medical Education, Faculty of Medicine, Tottori University, Yonago 680-8503, Japan

**Keywords:** prostaglandin E1, tumor-associated macrophage, phenotypic plasticity, prostaglandin receptor, TREM2, CXCR2

## Abstract

M2-like tumor-associated macrophages (TAMs) are a promising target for cancer immunotherapy, particularly for cancer patients who are refractory to current immune checkpoint inhibitors (ICIs). Previously, we showed that prostaglandin E1 (PGE1) enhances the expression of M1 markers, including HLA-DR, on macrophages and induces the M1 polarization of TAMs in vivo. This study investigated the pharmacological mechanisms by which PGE1 and its derivatives suppress the expression of M2 markers, including TREM2 and CXCR2. Macrophages were cultured in ultralow attachment dishes either alone or in combination with liver cancer cell lines to generate homospheroids or heterospheroids. Cell surface marker expression was assessed by flow cytometry. Compared with homospheroids, M2 marker expression on macrophages in heterospheroids was significantly increased, suggesting that heterospheroid culture promotes M2 polarization. PGE1 decreased M2 marker expression in heterospheroids more effectively compared with PGE2, PGE3, misoprostol, and 13,14-dihydro-15-keto-PGE1, whereas the suppressive effects of 15-keto- and 13,14-dihydro-PGE1s, and lubiprostone were comparable to that of PGE1. Pharmacological inhibition of prostaglandin receptors revealed that EP2 and EP4 receptors are involved in the PGE1-induced reprogramming of M2-like macrophages to M1 macrophages. In summary, PGE1 and its derivatives are promising TAM-targeting immunotherapeutics.

## 1. Introduction

Macrophages exhibit functional plasticity between the inflammatory and anti-tumor (M1) phenotype and the anti-inflammatory and protumor (M2) phenotype [1]. Tumor-associated macrophages (TAMs) are mostly inclined toward the M2-like phenotype and play an important role in cancer progression [1]. For example, TAMs promote T cell exhaustion and regulatory T cell differentiation and secrete protumor and immunosuppressive cytokines, such as IL-10, to establish an immunosuppressive microenvironment [2,3,4]. Moreover, TAMs are associated with the poor prognosis of patients with cancer because of their malignant functions in therapeutic resistance, angiogenesis, and metastasis [5,6,7,8]. These results indicate the potential of TAMs as an alternative therapeutic target to exhausted T-cell immunity, which is the primary target of current immune checkpoint inhibitors (ICIs).

The M2-like phenotype of TAMs is, in part, conferred by cancer cells through humoral factors and direct cell-to-cell contact [1,9]. These mechanisms, including their downstream pathways, are potential drug targets for reprogramming M2-like macrophages toward the M1 phenotype (herein referred to as TAM activators). However, to develop and assess TAM activators, an in vitro culture system is required that accurately reproduces the interaction between macrophages and cancer cells. We previously demonstrated that heterospheroids consisting of macrophages and liver cancer cells recapitulate some aspects of this cellular interaction in vitro and identified prostaglandin E1 (PGE1) as a TAM activator using heterospheroids [10,11]. In the heterospheroids, PGE1 increased the expression of M1 markers, such as HLA-DR and CD80, on macrophages. Moreover, PGE1 promoted M1-like polarization of TAMs, increased tumor-infiltrating CD8^+^ T cells, and enhanced the therapeutic efficacy of an anti-PD-1 antibody in a syngeneic mouse xenograft model [11]. While the therapeutic efficacy of PGE1 has been demonstrated in vitro and in vivo, its impact on M2 marker expression and the underlying mechanisms remain unclear.

Several studies suggest that it is difficult to detect expression of the widely used M2 markers, CD163 and CD206, in THP-1-derived macrophages [12,13,14]. Our experimental conditions also could not detect the upregulation of these M2 markers accompanied by M2 polarization [11]. Instead, we found that HX531, another TAM activator identified by our group and others [11,15], inhibited the transcription of other M2 markers in macrophages, including triggering receptor expressed on myeloid cells 2 (TREM2) and C-X-C motif chemokine receptor 2 (CXCR2) [11]. Importantly, TREM2 and CXCR2 directly promote the protumor functions of TAMs, and the inhibition of their expression and activity has been shown to suppress tumor growth and enhance the efficacy of tumor immune therapy [16,17,18,19]. Given these findings, TREM2 and CXCR2 are promising therapeutic targets for the development of TAM activators.

Therefore, in the present study, we determined the effects of PGE1 and its derivatives on M2 polarization by assessing TREM2 and CXCR2 macrophage surface expression in heterospheroids. We also identified the receptors involved in its mechanism of action using specific inhibitors for each prostaglandin receptor.

## 2. Materials and Methods

### 2.1. Materials

Epidermal growth factor (EGF), basic fibroblast growth factor (bFGF), macrophage colony-stimulating factor (M-CSF), and interferon γ (IFNγ) were obtained from Pepro Tech (Rocky Hill, NJ, USA). Allophycocyanin-conjugated HLA-DR (clone L243) and Alexa Fluor 647-conjugated TREM2 (clone: 237920R) antibodies were purchased from BioLegend (San Diego, CA, USA), while Alexa Fluor 647-conjugated CXCR2 antibody (clone: 5E8/CXCR2) was purchased from R&D Systems (Minneapolis, MN, USA). Lipopolysaccharide (LPS) was from Sigma-Aldrich (St. Louis, MO, USA). PGE1, PGE2, lubiprostone, misoprostol, TG4-155, E7046, and RO1138452 were purchased from Selleck (Houston, TX, USA). PGE3, 13,14-dihydro-PGE1 (D-PGE1), 13,14-dihydro-15-keto-PGE1 (DK-PGE1), 15-keto-PGE1 (K-PGE1), and AH6809 were purchased from Cayman Chemical (Ann Arbor, MI, USA). Phorbol 12-myristate 13-acetate (PMA) was purchased from Nacalai Tesque (Kyoto, Japan)

### 2.2. Cell Culture

HLF (human hepatocellular carcinoma), HuH6 (human hepatoblastoma), and THP-1 (human acute monocytic leukemia) cell lines were obtained from the Japanese Collection of Research Bioresources Cell Bank and the RIKEN Bioresource Center, respectively. HLF and HuH6 cells were maintained in DMEM (Shimadzu Diagnostics, Tokyo, Japan) supplemented with 10% FBS (Sigma-Aldrich). THP-1 cells were maintained in RPMI1640 (Shimadzu Diagnostics) supplemented with 10% FBS. Mycoplasma negativity was confirmed by PCR using VenorGeM Classic Mycoplasma Detection Kit (Minerva Biolabs GmbH, Berlin, Germany). These cell lines were authenticated by STR analysis (Fasmac, Kanagawa, Japan). In order to distinguish macrophages from liver cancer cells by flow cytometry, HLF and HuH6 cells were stably transfected with pAcGFP1-C1 (TaKaRa Bio, Kusatsu, Japan), followed by G418 (Nacalai Tesque) selection, and THP-1 cells were infected with a lentivirus expressing humanized Kusabira-Orange 1 (hKO-1). AcGFP- and hKO-1-positive clones were isolated by limited dilution and identified by fluorescent microscopy.

### 2.3. Preparation of THP-1-Derived Macrophages

hKO1-expressing THP-1 cells (3 × 10^6^ cells) were seeded into a 10 cm dish and incubated for 72 h in the presence of 200 nM PMA to induce differentiation into macrophages (day 0 to 3) (Figure S1a). The cells were further cultured in PMA-free RPMI1640 containing 10% FBS, which was replaced with fresh medium every day for three days to induce maturation (days 3 to 6). Although the expression levels of HLA-DR, TREM2, and CXCR2 varied during differentiation and maturation, THP-1 cells maintain their expression levels to some extent just before heterospheroid culture (Figure S1b–d).

### 2.4. Preparation of Primary Macrophages

The isolation of peripheral blood monocytes from a healthy male volunteer was conducted in accordance with both the Declaration of Helsinki and was approved by the Ethical Committee of Tottori University (22B016). Written informed consent was obtained from the donor before blood collection. 60 mL of peripheral blood with EDTA-2Na was centrifuged at 800× *g* for 15 min. After discarding the plasma, the buffy coat was recovered, diluted more than twice with RPMI1640 (FBS-free), and layered onto Monocytes Spin Medium (pluriSelect, Leipzig, Germany). Following centrifugation at 800× *g* for 20 min, the cells in the middle layer were recovered and washed twice with RPMI1640. The cells were then seeded into a 6-well plate and incubated at 37 °C in a 5% CO_2_ atmosphere for 1.5 h. After removing unattached cells by washing three times with PBS, the attached cells were incubated in RPMI1640 supplemented with 10% FBS, 4.5 g/L glucose, and 25 ng/mL M-CSF. Half of the medium was replaced with fresh medium every three days for 10 days to obtain mature macrophages. The cells were labeled with CellTracker CM-DiI Dye (Thermo Fisher Scientific, Waltham, MA, USA) prior to spheroid culture.

### 2.5. Spheroid Culture and Flow Cytometry

Cells were detached from the culture dish using accutase (Nacalai Tesque) for HLF and HuH6 cells and THP-1-derived macrophages, or using trypsin/EDTA (Nacalai Tesque) for primary macrophages. The macrophages (2.5 × 10^5^ cells) were thoroughly mixed either alone (homospheroids) or with 5 × 10^5^ HLF or HuH6 cells (heterospheroids) in Ham’s F-12 medium (Nacalai Tesque) supplemented with 20 ng/mL each of EGF and bFGF, and seeded in an ultra-low attachment 3.5 cm dish (Sumitomo Bakelite, Tokyo, Japan). Then, reagents were added to the cells at a concentration of 2 µM, and the same volume of dimethyl sulfoxide (DMSO) was added to the control cells, followed by three-day incubation. After dissociation of spheroids with accutase, the cells were incubated with 4 ng/mL of antibodies in 1% bovine serum albumin/PBS on ice for 30 min. Following filtration through a 30-µm mesh membrane (CS CRIE, Kyoto, Japan), the cells were analyzed by flow cytometer (CytoFLEX S, Beckman Coulter, Brea, CA, USA). The frequency of M1 or M2 marker-positive cells in hKO-1^+^ THP-1-derived macrophages or DiI^+^ primary macrophages (% positivity) was calculated by dividing the number of allophycocyanin- or Alexa Fluor 647-positive macrophages by the number of hKO-1^+^ or DiI^+^ macrophages. The expression levels of M1 and M2 markers were determined by the geometric mean (GeoMean; Π^1/n^, where Π is the product notation of fluorescent intensity and n is the number of macrophages) of allophycocyanin or Alexa Fluor 647 fluorescent intensity in macrophages. The gating strategy for analyzing hKO-1^+^ or DiI^+^ macrophages is shown in Figure S2.

### 2.6. Statistical Analysis

Data were analyzed using SPSS software (ver. 28.0, IBM, Armonk, NY, USA), and graphs were drawn using Excel software (ver. 16.87, Microsoft, Redmond, WA, USA). All experimental values were expressed as the mean ± standard deviation. Three or more independent samples for each experiment were analyzed as indicated in the figure legends. Differences between the two groups were assessed by Student’s *t*-test, while multiple comparisons were made by Bonferroni–Holm’s, Dunnett’s, or Tukey’s tests as indicated. A *p*-value less than 0.05 is considered as statistical significance.

## 3. Results

### 3.1. Promotion of M2 Polarization in Heterospheroids

To investigate the effect of HLF hepatocellular carcinoma cells on the M1 polarization of THP-1-derived macrophages, we treated homospheroids (containing only macrophages) and heterospheroids (containing both macrophages and HLF cells) with M1 inducers (IFNγ and LPS).

**Figure 1:**
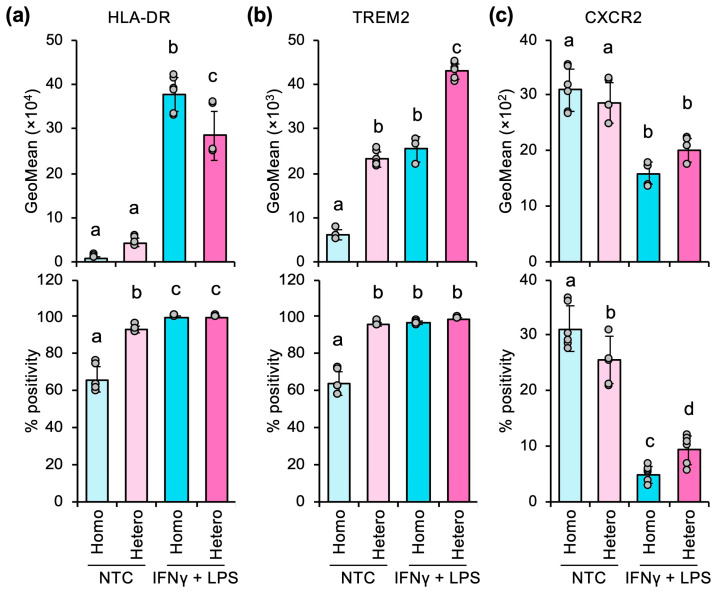
Heterospheroid culture suppresses M1 polarization and promotes M2 polarization. (**a**–**c**) The expression (geometric mean, GeoMean) and percent positive frequency (% positivity) of HLA-DR (**a**), TREM2 (**b**), and CXCR2 (**c**) in homospheroids (Homo; only hKO-1^+^ THP-1-derived macrophages) and heterospheroids (Hetero; hKO-1^+^ THP-1-derived macrophages and HLF hepatocellular carcinoma cells) that were untreated (non-treatment control; NTC) or treated with M1 inducers (20 ng/mL IFNγ and 10 ng/mL LPS) for three days, as determined by flow cytometry. Different letters indicate significant differences (*p* < 0.05, by Bonferroni-Holm’s test, *n* = 6).

In the absence of M1 inducers, the frequency of HLA-DR^+^ cells (% positivity) was significantly higher in heterospheroids than in homospheroids (Figure 1a). However, despite this difference in frequency, no significant difference in HLA-DR expression levels (GeoMean) was observed between homospheroids and heterospheroids. Moreover, it was observed that HLA-DR expression levels were markedly lower in the absence of M1 inducers than in their presence, suggesting that the presence of M1 inducers is crucial for effective M1 polarization, particularly in homospheroids. Notably, as previously shown [11], in the presence of M1 inducers, HLA-DR expression levels were significantly higher in homospheroids compared to those in heterospheroids (Figure 1a), suggesting that M1 polarization by M1 inducers is suppressed in heterospheroids.

TREM2 expression was increased in heterospheroids and further enhanced by M1 inducers (Figure 1b). CXCR2 expression tended to decrease in heterospheroids in the absence of M1 inducers (Figure 1c). In contrast to HLA-DR, CXCR2 expression was markedly downregulated by M1 inducers, whereas its downregulation was inhibited in heterospheroids (Figure 1c). Although their response to M1 inducers was different, these M2 markers were upregulated in homospheroids, suggesting that heterospheroid culture promotes M2 polarization.

### 3.2. Suppression of M2 Polarization by the PGE Subtypes

PGE1 is synthesized from dihomo-γ-linolenic acid by cyclooxygenases, whereas PGE2 and PGE3 are derived from arachidonic acid (AA) and eicosapentaenoic acid, respectively (Figure S3) [20]. Because AA is one of the most abundant polyunsaturated fatty acids in the cell membranes of mammals, PGE2 is synthesized more abundantly among the PGE subtypes during an inflammatory response; however, this preference is dependent upon dietary lipid composition [20,21].

**Figure 2:**
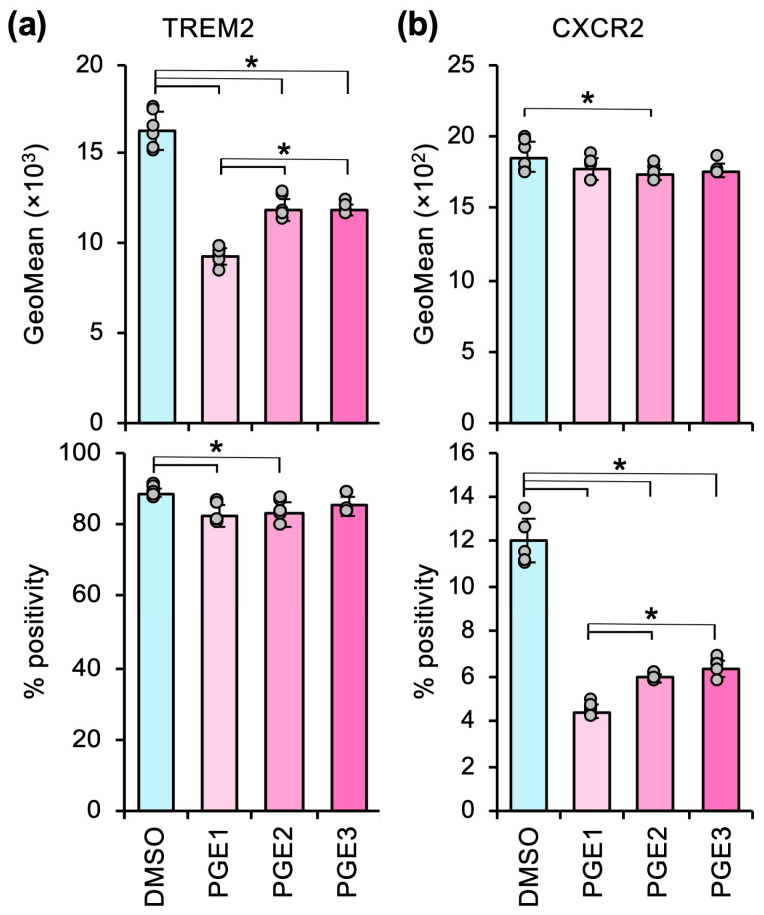
PGE1 and its subtypes counteract M2 polarization in heterospheroids containing hKO-1^+^ THP-1-derived macrophages and HLF hepatocellular carcinoma cells. (**a**,**b**) The expression (GeoMean) and percent positive frequency (% positivity) of TREM2 (**a**) and CXCR2 (**b**) in heterospheroids treated with DMSO or 2 µM of PGE1, PGE2, or PGE3 for three days, as determined by flow cytometry. *, *p* < 0.05 between the indicated groups by Tukey’s test (*n* = 6).

PGE1, PGE2, and PGE3 significantly suppressed TREM2 expression level (GeoMean) and CXCR2^+^ frequency (% positivity) in heterospheroids (Figure 2), which suggests that these PGE subtypes counteracted the M2 polarization induced by heterospheroids; however, PGE1 was the most potent in suppressing the M2 markers.

### 3.3. Suppression of M2 Polarization by PGE1 Metabolites

A substantial proportion of circulating PGE1 is rapidly metabolized to K-PGE1 during its first pass through the lung (Figure S4) [22,23]. Subsequently, DK-PGE1 is formed from K-PGE1 and is metabolized to D-PGE1 (Figure S4) [22,23]. However, because comparable pharmacological and clinical effects were observed following the intravenous or intra-arterial administration of PGE1 [24], the presence of biologically active metabolites has been suggested. Indeed, the anticoagulant effect of D-PGE1 is comparable to that of PGE1, whereas K- and DK-PGE1s are inactive [23,25].

**Figure 3:**
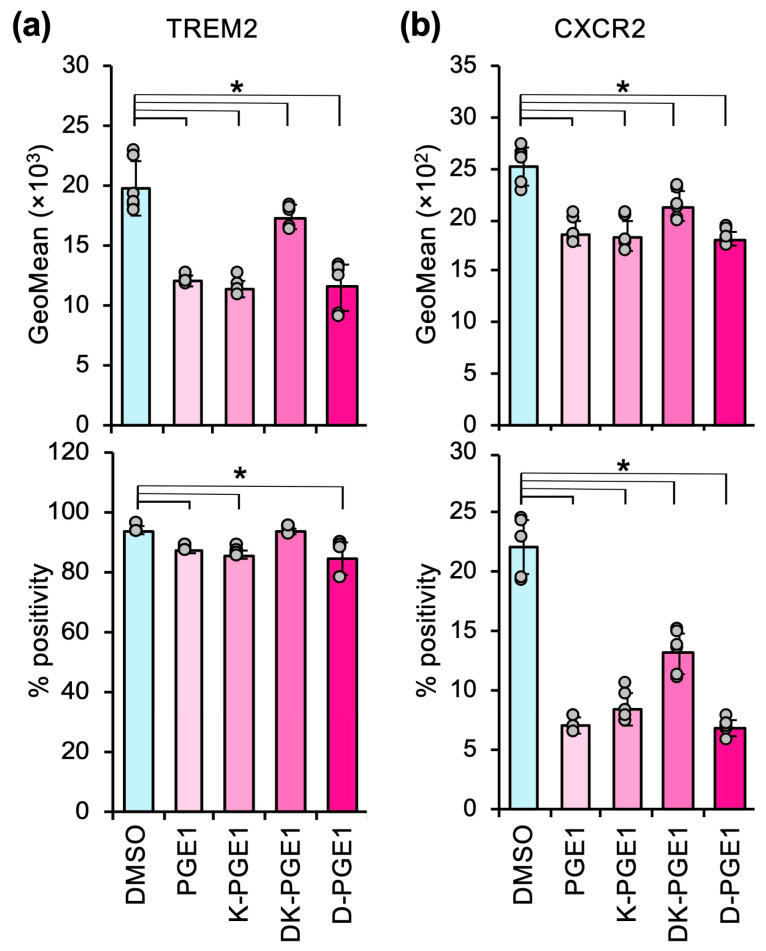
PGE1 metabolites counteract M2 polarization in heterospheroids containing hKO-1^+^ THP-1-derived macrophages and HLF hepatocellular carcinoma cells. (**a**,**b**) The expression (GeoMean) and percent positive frequency (% positivity) of TREM2 (**a**) and CXCR2 (**b**) in heterospheroids treated with DMSO or 2 µM of PGE1, 15-keto-PGE1 (K-PGE1), 13,14-dihydro-15-keto-PGE1 (DK-PGE1), or 13,14-dihydro-PGE1 (D-PGE1) for three days, as determined by flow cytometry. *, *p* < 0.05 compared to DMSO by Dunnett’s test (*n* = 6).

Based on these results, we determined the effects of these PGE1 metabolites on M2 polarization. As expected, M2 marker expression was decreased to a similar extent by PGE1 and D-PGE1 (Figure 3). However, unlike the anticoagulation effect, K-PGE1 exhibited similar suppressive effects as PGE1 and D-PGE1 (Figure 3). DK-PGE1 also suppressed M2 marker expression, although to a lesser extent compared with that of the other PGE1 metabolites (Figure 3). These results suggest that PGE1 and its metabolites suppress M2 polarization, albeit at varying levels.

### 3.4. Suppression of M2 Polarization by the PGE1 Derivatives

Lubiprostone and misoprostol are PGE1 derivatives that are used for the treatment of chronic idiopathic constipation and gastric ulcers, respectively. Lubiprostone is a bicyclic fatty acid; however, its monocyclic tautomer has a structure similar to PGE1 (Figure S5). Although its target is the ClC-2 chloride channel [26], lubiprostone also activates prostaglandin receptors [27,28,29,30]. Misoprostol is a methyl ester that is rapidly de-esterified by esterases to yield its biologically active form, misoprostol free acid (Figure S5) [31]. Thus, the affinity of misoprostol to prostaglandin receptors is much weaker compared with that of PGE1 [32].

**Figure 4:**
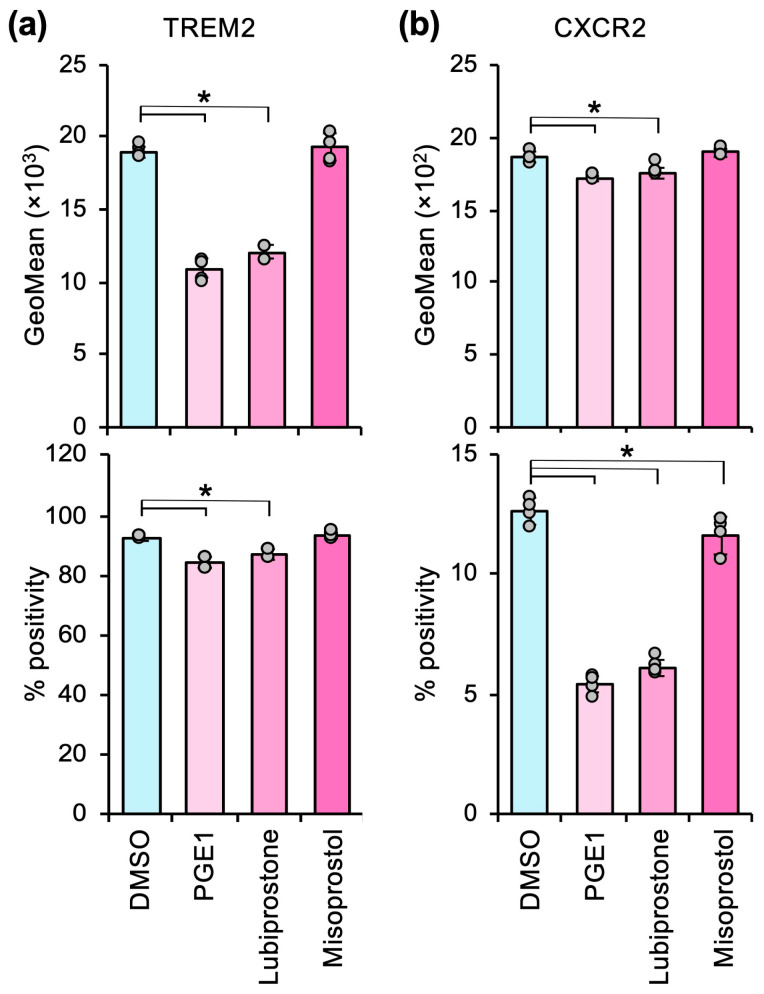
PGE1 derivatives counteract M2 polarization in heterospheroids containing hKO-1^+^ THP-1-derived macrophages and HLF hepatocellular carcinoma cells. (**a**,**b**) The expression (GeoMean) and percent positive frequency (% positivity) of TREM2 (**a**) and CXCR2 (**b**) in heterospheroids treated with DMSO or 2 µM of PGE1, lubiprostone, or misoprostol for three days, as determined by flow cytometry. *, *p* < 0.05 compared to DMSO by Dunnett’s test (*n* = 6).

Lubiprostone significantly suppressed M2 marker expression to a similar extent as PGE1 (Figure 4); however, misoprostol did not affect M2 marker expression. Therefore, lubiprostone, like PGE1, has the potential for drug repositioning as a cancer immunotherapeutic agent.

### 3.5. Identification of Prostaglandin Receptors Involved in the Mechanism of Action of PGE1

PGE1 binds to the EP1, EP2, EP3, EP4, and IP prostaglandin receptors [32]. Our transcriptome analysis of macrophages in heterospheroids revealed that these receptors were expressed at 3.24, 18.16, 0.22, 44.57, and 16.23 transcripts per million, respectively [11]. Therefore, we determined the effect of inhibiting these receptors, except for the EP3 receptor, on the activity of PGE1.

**Figure 5:**
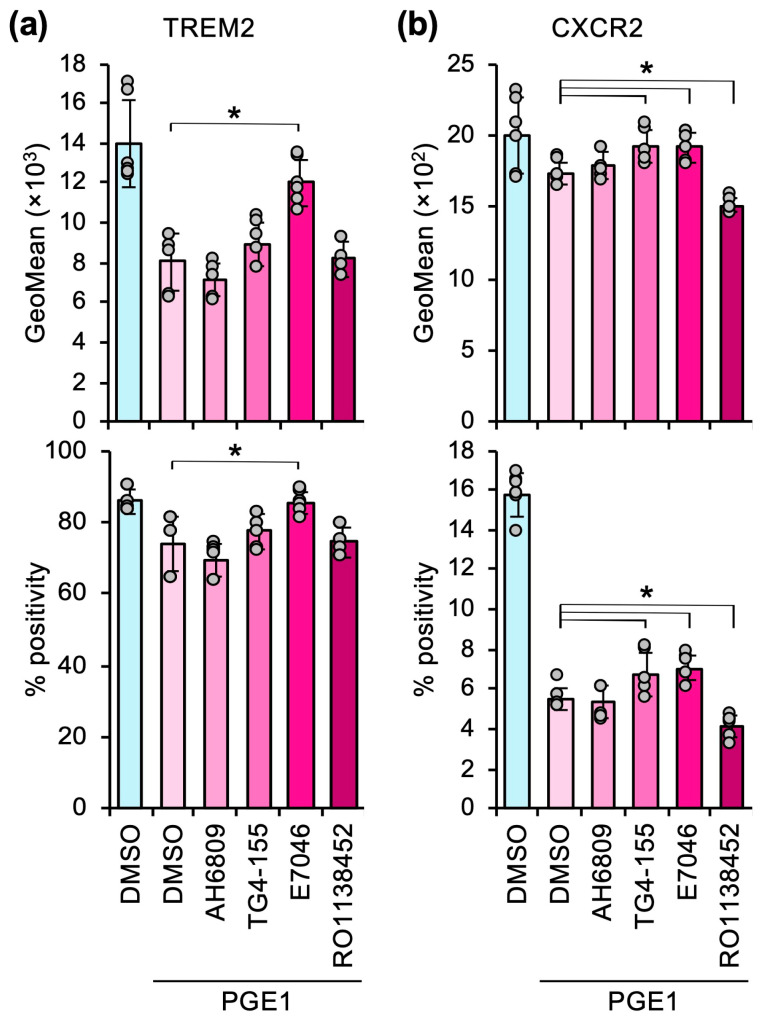
Prostaglandin receptors are involved in the effects of PGE1. (**a**,**b**) Heterospheroids containing hKO-1^+^ THP-1-derived macrophages and HLF hepatocellular carcinoma cells were treated with DMSO, PGE1 with DMSO, AH6809 (EP1 receptor antagonist), TG4-155 (EP2 receptor antagonist), E7046 (EP4 receptor antagonist), or RO1138452 (IP receptor antagonist) for three days. These agents were administered at a concentration of 2 µM. The expression (GeoMean) and percent positive frequency (% positivity) of TREM2 (**a**) and CXCR2 (**b**) were determined by flow cytometry. *, *p* < 0.05 compared to PGE1 alone by Dunnett’s test (*n* = 6).

As shown in Figure 5, AH6809 (EP1 receptor antagonist), TG4-155 (EP2 receptor antagonist), and RO1138452 (IP receptor antagonist) did not show any effect on the PGE1-induced downregulation of TREM2, whereas E7046 (EP4 receptor antagonist) significantly restored TREM2 expression. CXCR2 expression was significantly restored by TG4-155 and E7046 but further suppressed by RO1138452 (Figure 5). These results suggest that PGE1 suppresses TREM2 expression via the EP4 receptor and CXCR2 expression through the EP2 and EP4 receptors. Moreover, in opposition to these receptors, PGE1-mediated IP receptor activation may exert a positive effect on CXCR2 expression.

### 3.6. The Involvement of EP4 Receptor in the Action of PGE1

**Figure 6:**
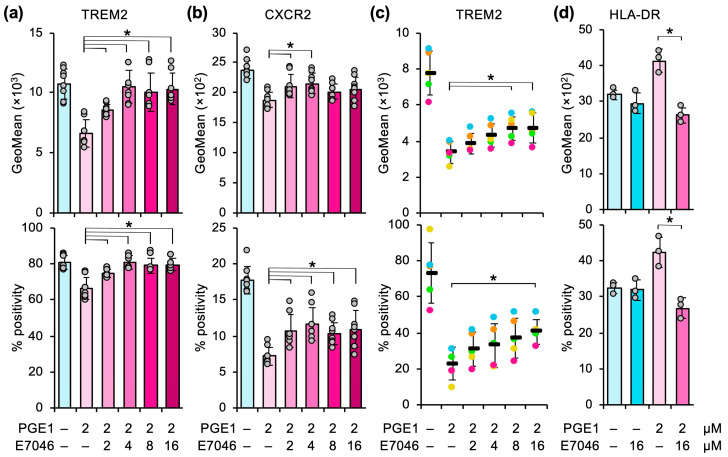
The effect of PGE1 is mediated, in part, by the EP4 receptor. (**a**,**b**) Heterospheroids containing hKO-1^+^ THP-1-derived macrophages and HLF hepatocellular carcinoma cells were treated with the indicated concentrations of PGE1 and E7046 for three days. The expression (GeoMean) and percent positive frequency (% positivity) of TREM2 (**a**) and CXCR2 (**b**) on THP-1-derived macrophages were determined by flow cytometry. *, *p* < 0.05 compared to PGE1 alone by Dunnett’s test (*n* = 8). (**c**) Heterospheroids containing DiI^+^ human peripheral monocyte-derived macrophages and HLF cells were treated with the indicated concentrations of PGE1 and E7046 for three days. The expression and percent positive frequency of TREM2 on primary macrophages were determined by flow cytometry. Each batch is shown in a different color. *, *p* < 0.05 compared to PGE1 alone by Dunnett’s test (*n* = 5). (**d**) Heterospheroids containing hKO-1^+^ THP-1-derived macrophages and HLF hepatocellular carcinoma cells were treated with the indicated combinations of PGE1 (2 µM) and E7046 (16 µM) for three days. The GeoMean and % positivity of HLA-DR on THP-1-derived macrophages were determined by flow cytometry. *, *p* < 0.05 between the absence and presence of E7046 by Student’s *t*-test (*n* = 3).

Because the EP4 receptor is involved in the PGE1-induced downregulation of TREM2 and CXCR2 (Figure 5), we determined the effect of E7046 on the activity of PGE1 in heterospheroids using THP-1-derived macrophages. The effect of PGE1 on TREM2 expression was abrogated by ≥4 µM E7046 (Figure 6a), whereas CXCR2 expression was not completely restored, even at 16 µM (Figure 6b).

Because PGE1 suppresses TREM2 expression in human peripheral monocyte-derived macrophages [11], we determined whether the EP4 receptor is involved in the effect of PGE1 in primary macrophages. Of note, the cell surface expression of CXCR2 was not detected under our experimental conditions. PGE1 suppressed TREM2 expression, whereas EP7046 significantly, but partially, restored TREM2 expression at concentrations of ≥8 µM (Figure 6c). Although the mechanisms of action are different between THP-1-derived and primary macrophages, PGE1 suppresses M2 polarization through the EP4 receptor as well as other pathways, such as the EP2 receptor.

We finally examined the involvement of the EP4 receptor in PGE1-induced M1 polarization in THP-1-derived macrophages. Increased HLA-DR expression in the presence of PGE1 was completely abrogated by E7046, suggesting that PGE1 induces HLA-DR expression solely via the EP4 receptor in THP-1-derived macrophages (Figure 6d). These findings were also observed in heterospheroids consisting of another liver cancer cell line, HuH6, instead of the HLF cell line (Figure S6).

## 4. Discussion

We have demonstrated that PGE1 induces M1-like reprogramming of TAMs in a syngeneic liver cancer transplantation model, resulting in suppressed tumor growth and enhanced anti-PD-1 antibody efficacy [11]. In the present study, we investigated the specific pathway through which PGE1 exerts its reprogramming effect on macrophages. Our findings indicate that EP2 and EP4 prostaglandin receptors play a crucial role in this process, highlighting the potential for a targeted therapeutic strategy. However, further in vivo studies are necessary to confirm the therapeutic relevance of these receptors in PGE1’s action. Given the study’s primary goal to clarify the pharmacological mechanism of PGE1 in reprogramming macrophages, we focused on M2 marker expression. However, we did not explore macrophage functions, which could provide further insight into the pharmacological action of PGE1. Future studies should precisely explore the effects of PGE1 and its receptors on the anti-tumor functions of macrophages.

Heterospheroid cultures have some advantages over monolayer and homospheroid cultures. Cancer cells instruct surrounding stromal cells in the tumor tissue to establish protumor niches through humoral factors and direct cell-to-cell contact. These cellular communications may be reproduced using in vitro heterospheroid culture. In addition, three-dimensional culture can recapitulate the features observed in tumor tissue, such as drug resistance, metastatic potential, metabolic reprogramming, and cancer stemness, compared with monolayer culture. Consistent with these findings, we demonstrated that M1 marker expression upregulated by M1 inducers was significantly suppressed in heterospheroids, whereas higher expression of M2 markers was observed (Figure 1). This suggests that heterospheroid culture also reproduces cancer cell-induced M2-like polarization of TAMs.

By taking these advantages of the heterospheroid culture, we demonstrated that PGE1, PGE2, and PGE3 suppressed M2 marker expression (Figure 2). We and others have demonstrated that PGE1 and PGE3 enhance the antitumor phenotype of TAMs [11,33]. In contrast, PGE2 is a well-known immune suppressor that compromises antitumor immunity on cancer cells [34], T cells [35,36], natural killer cells [37,38], dendritic cells [38], monocytes [39,40], and macrophages [41,42], mainly through EP2/EP4 receptors. However, a transcriptome analysis performed by Rosner et al. [43] revealed that activation of the PGE2-EP2/EP4 signaling pathway in CD8+ tumor-infiltrating lymphocytes was associated with improved recurrence-free survival in non-small cell lung cancer patients. The clinical and biological significance of this observation, including its causal relationship, remains to be clarified; however, the PGE2-EP2/EP4 signaling pathway is not always associated with an immunosuppressive microenvironment.

In our previous study, we demonstrated that once-daily intraperitoneal administration of PGE1 significantly suppressed tumor growth in a syngeneic mouse xenograft model [11]. However, intravenously administered PGE1 is rapidly eliminated from the human body, with half-lives of 0.17 min in the α phase and 8.2 min in the β phase [44]. Because D-PGE1 exerts anticoagulant activity similar to that of PGE1 [23,25], PGE1 metabolites may also contribute to our in vivo observations. Consistent with this hypothesis, we demonstrated that K-PGE1 suppresses M2 polarization with a similar activity to that of PGE1 and D-PGE1 (Figure 3). EP2/EP4 receptors are Gs-coupled receptors that produce cAMP upon ligand binding [45], and D-PGE1 activates adenylyl cyclase via EP2/EP4 receptors [46,47]. Therefore, similar to PGE1, D-PGE1 may suppress M2 marker expression through EP2/EP4 receptors. The mechanism of action of K-PGE1 remains to be elucidated; however, PGE1 and PGE2 inhibit human platelet aggregation via the IP and EP4 receptor [48], whereas K-PGE1 exhibits minimal anticoagulant activity (less than 1/100 that of PGE1 [23]). This suggests that K-PGE1 acts through distinct mechanisms independent of the EP4 receptor. On the other hand, from a clinical viewpoint, K-PGE1 may be a safer alternative to PGE1 because PGE1 is accompanied by bleeding. However, because K-PGE1 is ultimately metabolized to D-PGE1 with anticoagulant activity, a detailed study of its metabolic profile is warranted for the development of novel immune therapeutics based on K-PGE1.

Similar to K-PGE1, lubiprostone is also a promising candidate for cancer immunotherapy targeting macrophages because it effectively suppresses M2 marker expression (Figure 4) and exhibits no anticoagulant activity [49]. Moreover, the clinical application of lubiprostone for treating drug-induced constipation in cancer patients has been established without serious adverse events [50,51]. Misoprostol failed to suppress M2 marker expression under our experimental conditions; however, the efficacy of this pro-drug should be examined in vivo because the free acid of misoprostol is relatively stable compared with PGE1, with half-lives of 1.7 h in the α phase and 157 h in the β phase [31].

To rule out the possible cytotoxicity of PGE1 and its derivatives, we determined the frequency of hKO-1^+^ cells (macrophages) in viable cells (cancer cells and macrophages) by flow cytometry. M1 inducers increased the frequency (Figure S7a), while PGE1 and its derivatives tended to decrease it (Figure S7b–f). In contrast, neither PGE1 nor E7046 changed the frequency of primary macrophage (Figure S7g). These results suggest that our findings are not due to the cytotoxic effects of PGE1 and its derivatives.

Although THP-1 cells are widely used as an in vitro model for biological and pathological studies of macrophages, they differ from primary or in vivo macrophages in some aspects [12,13,14]. Our results indicate that PGE1 suppresses TREM2 expression solely through the EP4 receptor in THP-1-derived macrophages, whereas in primary macrophages, EP4 and other pathways may be involved (Figure 6). One candidate is the EP2 receptor, which is involved in the suppression of CXCR2 by PGE1 in THP-1-derived macrophages. Other putative mechanisms include prostaglandin receptor-independent pathways. For example, PGE1 directly binds to NURR1, thereby activating its transactivation activity [52]. Moreover, NURR1 is induced by inflammatory stimuli and counteracts the inflammatory response in macrophages derived from human peripheral monocytes [53]. Prostanoids, including PGE1, exhibit pleotropic physiological functions; however, their underlying mechanisms remain unclear. Therefore, a comprehensive study is needed to determine how PGE1 and its derivatives reprogram the macrophage phenotype.

## 5. Conclusions

PGE1 inhibits M2 polarization of macrophages via the EP2 and EP4 receptors. Moreover, K- and D-PGE1s, as well as lubiprostone, exhibit similar suppressive activity. PGE1 and its derivatives may represent novel immunotherapeutics for targeting TAMs.

## 6. Patents

A patent for PGE1 as a macrophage activator was submitted.

## Data Availability

The raw data supporting the conclusions of this article will be made available by the authors on request.

## Supplementary Materials

The following supporting information can be downloaded at: https://www.mdpi.com/article/10.3390/cells14241992/s1, Figure S1: The expression of M1 and M2 macrophage polarity markers on THP-1 cell surface during macrophage differentiation and maturation; Figure S2: Gating strategy for flowcytometry; Figure S3: Biosynthetic pathway for the PGE subtypes; Figure S4: Metabolic pathway for PGE1; Figure S5: Chemical structures of lubiprostone (bicyclic and monocyclic forms) and misoprostol; Figure S6: PGE1-induced macrophage polarization in heterospheroids consisting of HuH6 cells; Figure S7: The frequency of macrophages to viable cells.

## Abbreviations

The following abbreviations are used in this manuscript:

AA	Arachidonic acid
bFGF	Basic fibroblast growth factor
CXCR2	C-X-C motif chemokine receptor 2
D-PGE1	13,14-Dihydro-prostaglandin E1
DK-PGE1	13,14-Dihydro-15-keto-prostaglandin E1
DMSO	Dimethyl sulfoxide
EGF	Epidermal growth factor
GeoMean	Geometric mean
ICI	Immune checkpoint inhibitor
IFNγ	Interferon γ
K-PGE1	15-Keto-prostaglandin E1
LPS	Lipopolysaccharide
M-CSF	Macrophage colony-stimulating factor
PGE1	Prostaglandin E1
PMA	Phorbol 12-myristate 13-acetate
TAM	Tumor-associated macrophage
TREM2	Triggering receptor expressed on myeloid cells 2
