# Supplementary material for: PGE1 Suppresses the Expression of M2 Markers on Macrophages Through Prostaglandin Receptors
Source: Cells. 2025 Dec 15;14(24):1992. doi: 10.3390/cells14241992 (PMC12731825; doi:10.3390/cells14241992)
Supplement: Supplementary file 1 [file cells-14-01992-s001.zip › cells-4048228-supplementary.pdf]

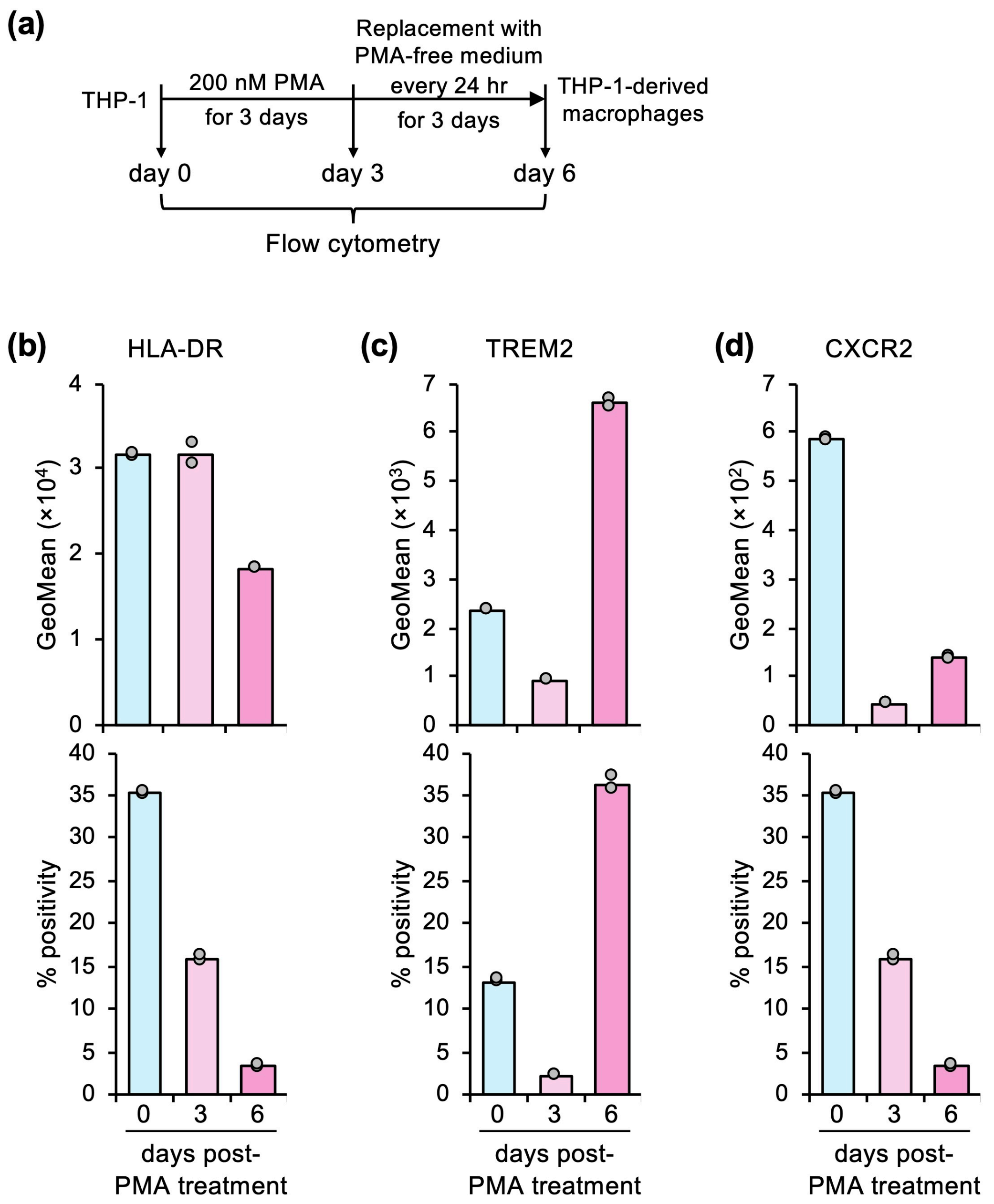

Figure S1. The expression of M1 and M2 macrophage polarity markers on THP-1 cell surface during macrophage differentiation and maturation. (**a**) Preparation of THP-1-derived macrophages. Flow cytometry analysis was performed at day 0 (before differentiation), day 3 (after differentiation), and day 6 (after maturation). PMA, phorbol 12-myristate 13-acetate. (**b**–**d**) The expression (GeoMean) and percent positive frequency (% positivity) of HLA-DR (**b**), TREM2 (**c**), and CXCR2 (**d**) on the cell surface at 0, 3, and 6 days post-PMA treatment. n = 2.

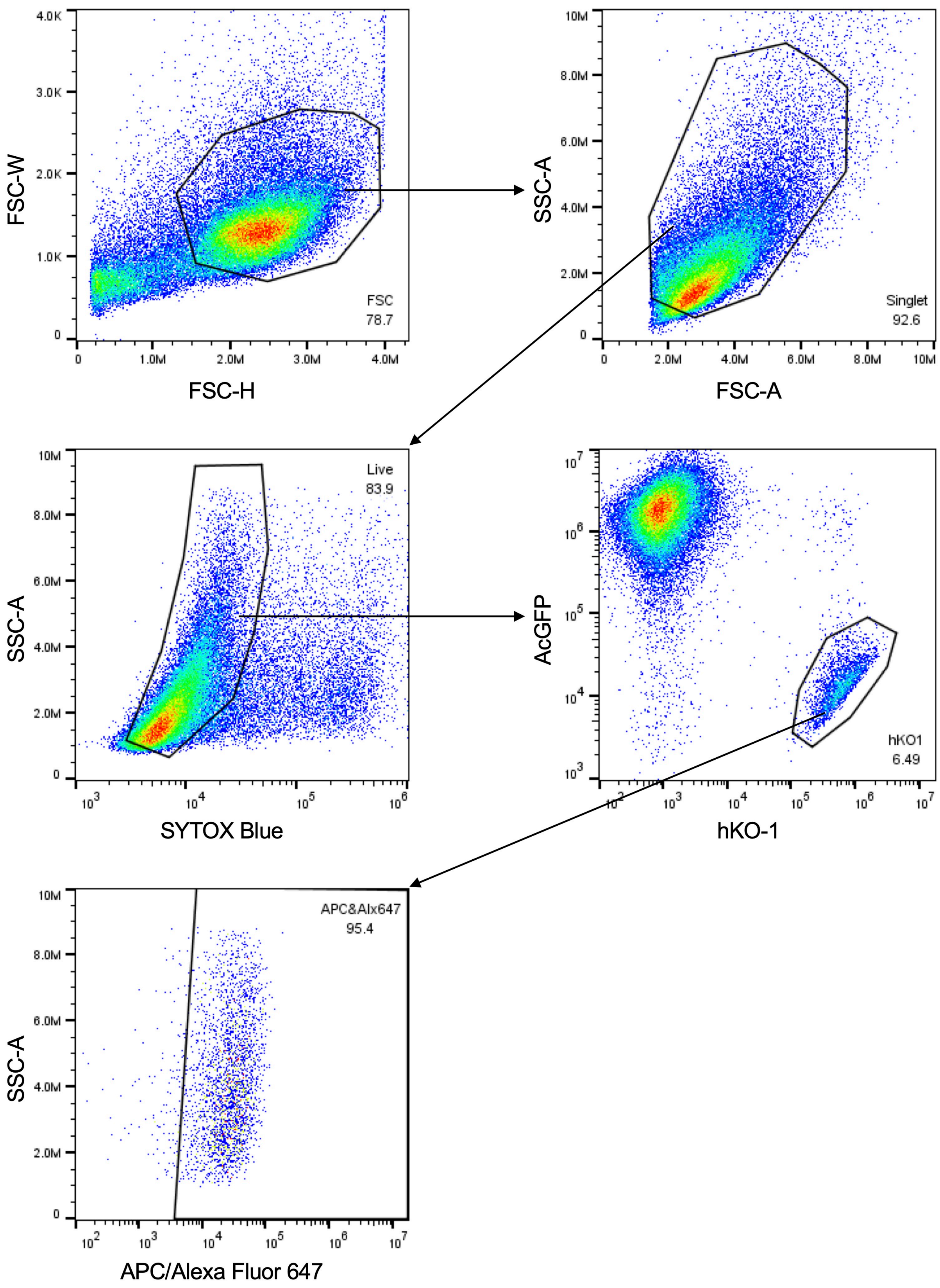

Figure S2. Gating strategy for flow cytometry analysis. The analyses were performed by CytoFLEX S.

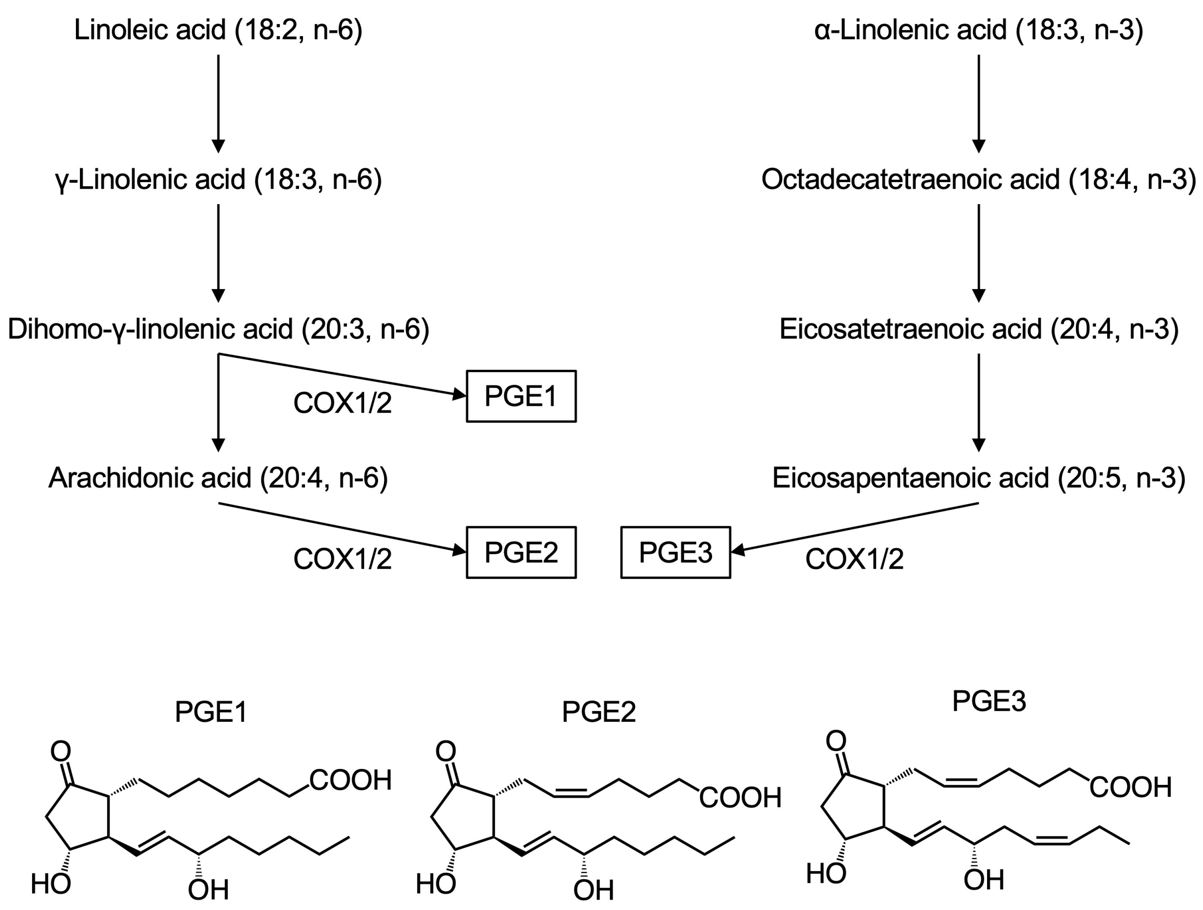

Figure S3. Biosynthetic pathway for the PGE subtypes. PGE1, PGE2, and PGE3 are synthesized from dihomo-γ-linolenic acid, arachidonic acid, and eicosapentaenoic acid, respectively, by cyclooxygenases 1 and 2 (COX1/2).

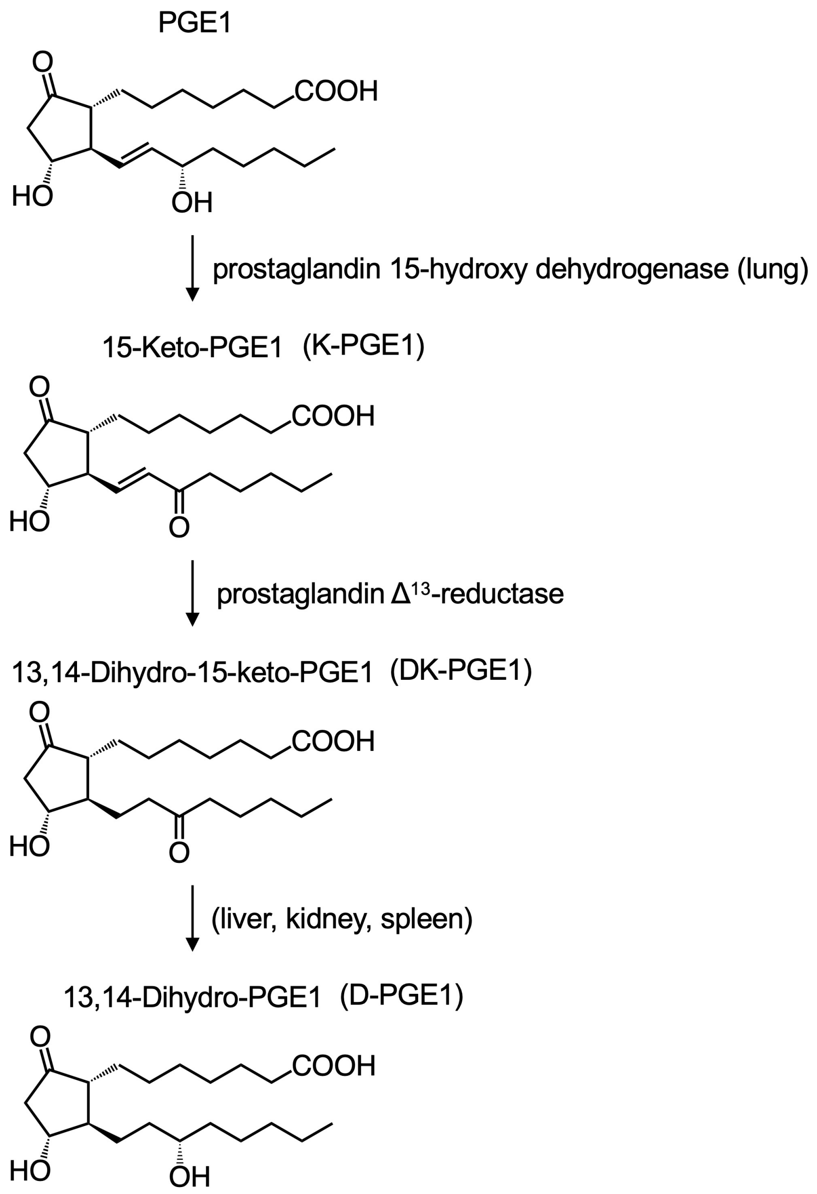

Figure S4. Metabolic pathway for PGE1. PGE1 is rapidly metabolized to D-PGE1 in the body. Details are described in the text.

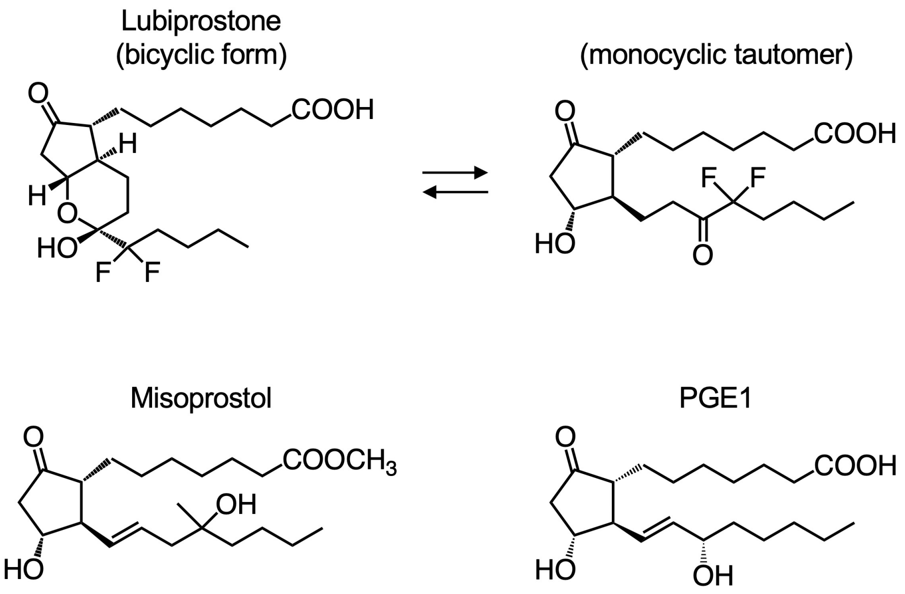

Figure S5. Chemical structures of lubiprostone (bicyclic and monocyclic forms) and misoprostol. Misoprostol and lubiprostone, clinically approved PGE1 derivatives, possess chemical structures similar to PGE1.

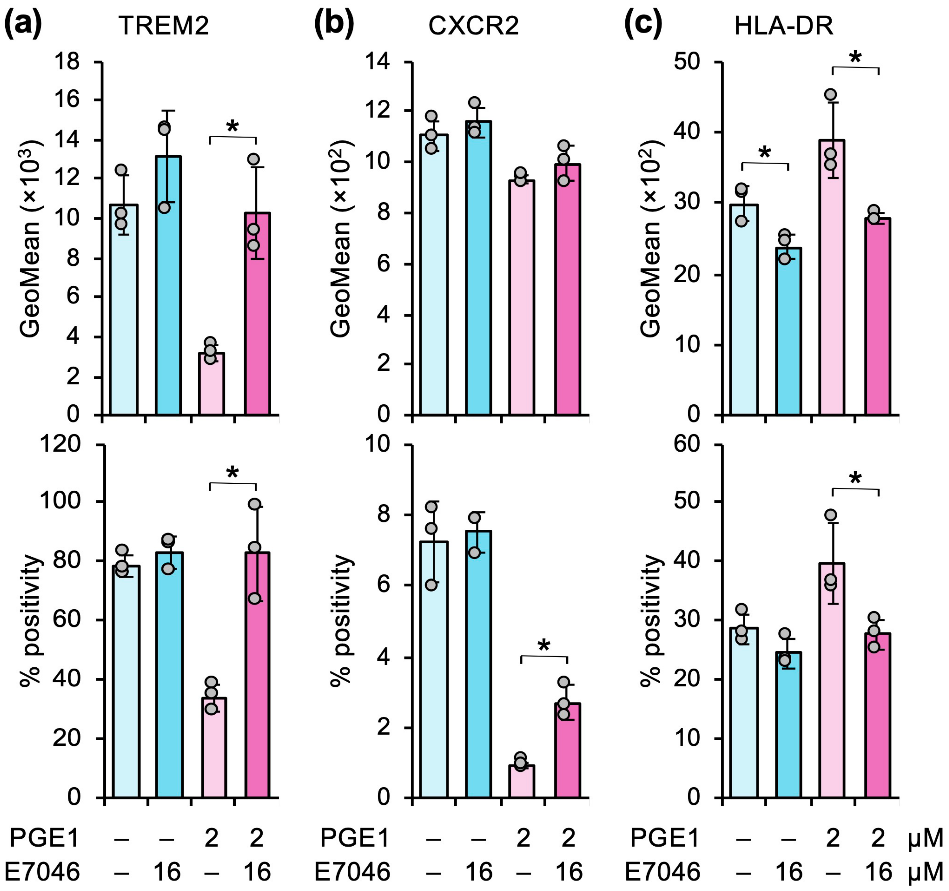

Figure S6: PGE1-induced macrophage polarization in heterospheroids consisting of HuH6 cells. The expression (GeoMean) and percent positive frequency (% positivity) of TREM2 (**a**), CXCR2 (**b**), and HLA-DR (**c**) on THP-1-derived macrophages in heterospheroids, as determined by flow cytometry. Heterospheroids containing hKO-1$^+$ THP-1-derived macrophages and HuH6 hepatoblastoma cells were treated with the indicated combinations of PGE1 (2 µM) and E7046 (16 µM) for three days. *, $P < 0.05$ between the absence and presence of E7046, by Student's *t*-test (n = 3).

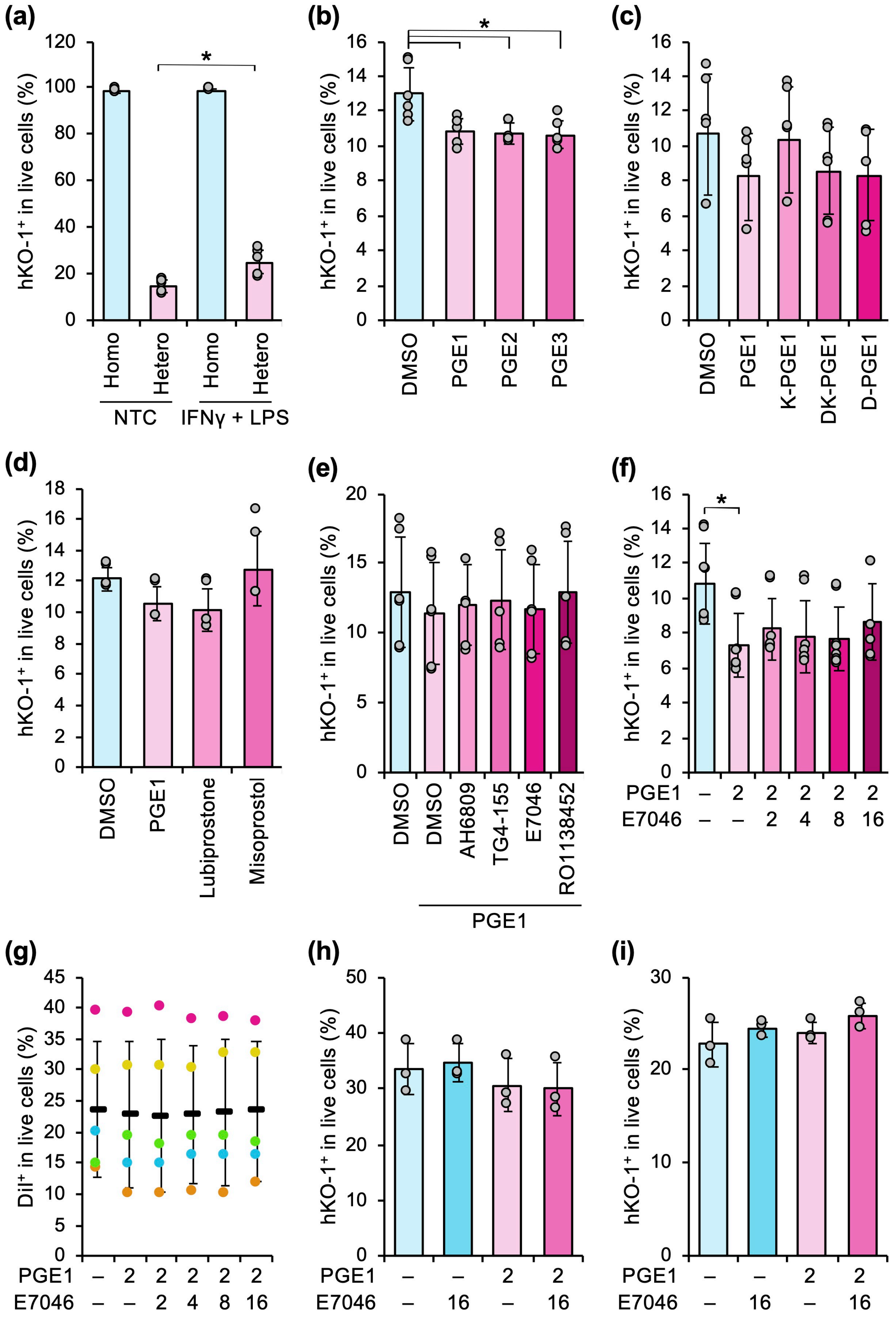

Figure S7. The frequency of macrophages to viable cells. The frequency of $KO1^+$ or DiI^+ macrophages in viable cells (macrophage and cancer cells [a–h, HLF cells; i, HuH6cells]) was determined by flow cytometry. *, $P < 0.05$ compared to non-treatment control (DMSO alone) by Student's *t*-test (a) or Dunnett's test (b–i) (n = 3–8).
